# Umbilical Cord Blood Bilirubin, Albumin, Reticulocyte Count, and Nucleated Red Blood Cells to Predict Subsequent Hyperbilirubinemia in Term Neonates: A Prospective Observational Study

**DOI:** 10.7759/cureus.37598

**Published:** 2023-04-14

**Authors:** Sadgunraju Chakrahari, Mallanagouda Patil, Hidaytullah R Bijapure

**Affiliations:** 1 Pediatrics, Shri B. M. Patil Medical College Hospital and Research Centre, BLDE (Deemed to be University), Vijayapur, IND; 2 Pediatric Medicine, Shri B. M. Patil Medical College Hospital and Research Centre, BLDE (Deemed to be University), Vijayapur, IND

**Keywords:** cord nucleated red blood, serum bilirubin, cord reticulocyte count, cord albumin, cord bilirubin

## Abstract

Introduction: Hyperbilirubinaemia is one of the most important causes of re-admission in the early neonatal period. The socioeconomic factors are one of the most common reasons for early discharge in a developing country like India.

Objectives: This study aims to evaluate and analyze the statistical correlation of umbilical cord blood bilirubin, albumin, nucleated red blood cells (nRBC), and reticulocyte count as early predictors of neonatal hyperbilirubinemia.

Method: A prospective observational study was conducted from November 2015 to April 2017 in a tertiary care hospital in North Karnataka, India. Umbilical cord blood was collected at birth for analysis of bilirubin, albumin, reticulocyte count, and nRBC in term neonates. Total serum bilirubin (TSB) levels were estimated using the VITROS BuBc Slide method at 72 hours of life. Data were analyzed using SPSS version 23 (IBM Corp., Armonk, NY).

Results: A total of 200 term neonates were enrolled in the study, out of which 123 completed follow-ups. Of the 66 newborns who had cord bilirubin levels ≥1.75 mg/dl, 23 (34.8%) developed hyperbilirubinemia after 72 hours of life, whereas 10 of the 57 newborns (17.5%) whose cord bilirubin levels <1.75 mg/dl developed hyperbilirubinemia after 72 hours of life. Cord blood albumin of ≥3.75 g/dl was seen in 93 neonates, of which 18 (19.4%) developed hyperbilirubinemia after 72 hours of life and 15 (50%) with <3.75 g/dl developed hyperbilirubinemia after 72 hours of life. Cord reticulocyte count ≥4.95% was seen in 54 neonates; 20 (37.03%) developed hyperbilirubinemia, whereas in 69 neonates with <4.95%, 13 (18.84%) developed hyperbilirubinemia after 72 hours of life. Of the 62 neonates who had cord nRBC ≥3.5%, 28 (45.2%) neonates developed hyperbilirubinemia after 72 hours of life, whereas 5 of the 61 neonates (8.19%) with cord nRBC <3.5% developed hyperbilirubinemia after 72 hours of life.

Conclusions: Cord blood bilirubin, albumin, reticulocyte counts, and nucleated RBC can be used as predictors of subsequent neonatal hyperbilirubinemia.

## Introduction

Hyperbilirubinemia is one of the most frequent causes of readmission in developing nations during the early newborn period. Hyperbilirubinemia often appears on days 2-4 of life in 50% of term and 80% of preterm neonates [1], and it usually spontaneously resolves on its own in one to two weeks [2]. In modern clinical practice, healthy-term neonates who have had a normal vaginal birth are allowed to go home sooner for various reasons, including financial constraints, social reasons like an early naming ceremony, or medical ones to protect them from nosocomial infections, which makes it challenging to identify, monitor, and treat hyperbilirubinemia in its early stages [3]. According to the American Academy of Pediatrics recommendations, babies discharged from the hospital within 48 hours of birth should be monitored between 48 and 72 hours to rule out severe neonatal jaundice and other issues [4].

Early discharge is most concerning because of bilirubin-induced brain damage in healthy-term neonates, even in the absence of hemolysis. Although it is unknown what total serum bilirubin (TSB) level causes an icteric neonate to develop kernicterus, a bilirubin level above 20 mg/dl has been linked to the emergence of kernicterus. It has been associated with severe forms of brain damage. Physical examination is unreliable in estimating serum bilirubin levels [5]. There is still no accurate method for estimating the risk of bilirubin-dependent brain damage that has been clinically tested [6, 7].

Only a few predictors of newborn jaundice have been explored to date, including alpha-fetoprotein, cord blood albumin, cord blood bilirubin, and the cord blood albumin to bilirubin ratio. We can efficiently design and perform the follow-up of the high-risk groups by predicting the neonates who will experience significant neonatal jaundice after delivery. By doing this, early therapy might be initiated, lowering the possibility of bilirubin-dependent brain damage.

Very few studies use cord bilirubin, albumin, nucleated red blood cells (nRBC), and reticulocyte count as predictors for hyperbilirubinemia. Hence, our study aimed to use all these factors to predict subsequent hyperbilirubinemia in neonates.

## Materials and methods

Study design and setting

A prospective observational and analytical study was conducted in the Department of Pediatrics at a tertiary care hospital in North Karnataka between November 2015 and April 2017.

Study participants

Healthy-term neonates fulfilling inclusion and exclusion criteria were included in the study.

**Table 1:** Inclusion and exclusion criteria

Inclusion criteria	Exclusion criteria
Term babies	Rh incompatibility
Birth weight >2500 g	ABO incompatibility
Apgar ≥7 and ≥9 at 1 min and 5 min	Neonatal sepsis
	Birth asphyxia
	Pathological jaundice
	Major congenital anomaly

The sample size was calculated using a previous study with an 87% sensitivity of cord albumin levels of <2.5 g/dl in predicting hyperbilirubinemia in neonates [8]. Assuming the prevalence of hyperbilirubinemia to be 50% in term neonates [1], 145 participants are required to detect a sensitivity of 75% with a precision of 0.1% and a 95% confidence level. Considering the 25% dropout and the follow-up for a second blood sample, a 194 sample size was determined. To round up, we decided to enroll 200-term neonates [9]. Following delivery, 200 neonates were enrolled in the study using the convenience sampling method.

Data collection

After enrolling in the study, their umbilical cords were double-clamped and transected within 10 seconds. Samples of cord bilirubin, albumin, reticulocyte count, and nRBC were collected. These neonates were monitored every morning after birth using the Kramer index to identify the development of jaundice. At 72 hours of life, serum bilirubin was estimated using a peripheral venous sample and the VITROS BuBc Slide method. Babies with serum bilirubin levels ≥12 mg/dl were followed up clinically in the ward until discharge or the seventh day of life, in accordance with the neonatal intensive care unit (NICU) protocol for treatment and follow-up of significant jaundice. Neonates who were discharged early from the hospital were called in at 72 hours of life for clinical assessment of clinically significant jaundice, serum bilirubin estimates were repeated, and management was performed according to protocol.

Statistic analysis

All characteristics were summarized descriptively. The summary statistics of mean and standard deviation (SD) were used for continuous variables. The number and percentage were used in the data summaries for categorical data. The difference in the means of the analysis variables between the two independent groups was tested by an unpaired t-test. The difference in the means of the analysis variables between more than two independent groups was tested by ANOVA and the F test of equality of variance. Bivariate correlation analysis using Pearson's correlation coefficient (r) was used to test the strength and direction of relationships between the interval levels of variables. A ROC analysis for sensitivity and specificity was done to check relative efficiency. If the p-value was <0.05, then the results were considered to be statistically significant; otherwise, they were considered not to be statistically significant. Data were analyzed using SPSS software v.23.0 (IBM Corp., Armonk, NY) and Microsoft Office.

Ethical consideration

Study approval was obtained from the Institutional Ethics Committee of Shri B. M. Patil Medical College and Research Centre, BLDEDU, Vijayapura, India, with IEC No/58/2015 dated November 20, 2015. Written informed consent was obtained from the mothers of the neonates enrolled in the study.

## Results

The total number of neonates in whom cord albumin, bilirubin, nRBC, and reticulocyte count were estimated was 200. Seventy-seven neonates who were discharged early from the hospital and did not turn up for the follow-up to re-estimate serum bilirubin at 72 hours were excluded from the study. The total number of neonates included in the study was 123. Baseline characteristics are mentioned in Table 2.

**Table 2:** Baseline characteristics (n=123).

Variable	Serum bilirubin ≤12 mg/dl	Serum bilirubin >12 mg/dl	p-value
Male	51	20	0.161
Female	43	9	
Mean birthweight (g)	2894.8	2942.7	0.417
Cesarean section	24	4	0.188
Normal delivery	70	25	

Out of 66 newborns who had cord bilirubin levels ≥1.75 mg/dl, 23 (34.8%) developed hyperbilirubinemia after 72 hours of life, whereas of the 57 newborns who had cord bilirubin levels <1.75 mg/dl, 10 (17.5%) developed hyperbilirubinemia after 72 hours of life (Table 3).

**Table 3:** Cord bilirubin and serum bilirubin levels (n=123) The p-value is 0.031*, which is statistically significant.

Bilirubin in cord blood (mg/dl)	Serum bilirubin after 72 hours ≥12 mg/dl n (%)	Serum bilirubin after 72 hours <12 mg/dl n (%)	p-value
≥1.75 (n=66)	23 (34.8)	43 (65.2)	0.031*
<1.75 (n=57)	10 (17.5)	47 (82.5)	
Total	33	90	

Out of 93 newborns who had cord albumin levels ≥3.75 g/dl, 18 (19.4%) developed hyperbilirubinemia after 72 hours of life, whereas of the 30 newborns who had cord albumin levels <3.75 g/dl, 15 (50%) developed hyperbilirubinemia after 72 hours of life (Table 4).

**Table 4:** Cord albumin and serum bilirubin levels (n=123) The p-value is 0.001*, which is statistically significant.

Albumin in cord blood (g/dl)	Serum bilirubin after 72 hours ≥12 mg/dl, n (%)	Serum bilirubin after 72 hours <12 mg/dl, n (%)	p-value
≥3.75 (n=93)	18 (19.4)	75 (80.6)	0.001*
<3.75 (n=30)	15 (50.0)	15 (50.0)	
Total	33	90	

Of the 54 newborns who had cord reticulocyte counts ≥4.95%, 20 (37.0%) developed hyperbilirubinemia after 72 hours of life, whereas of the 69 who had cord reticulocyte counts <4.95%, 13 (18.8%) developed hyperbilirubinemia after 72 hours of life (Table 5).

**Table 5:** Cord reticulocyte counts and serum bilirubin levels (n=123) The p-value is 0.024*, which is statistically significant.

Reticulocyte counts in cord blood %	Serum bilirubin after 72 hours ≥12 mg/dl n (%)	Serum bilirubin after 72 hours <12 mg/dl n (%)	p-value
≥4.95 (n=54)	20 (37.0)	34 (63.0)	0.024*
<4.95 (n=69)	13 (18.8)	56 (81.2)	
Total	33	90	

Of the 62 newborns who had cord nRBC ≥ 3.5%, 28 (45.2%) developed hyperbilirubinemia after 72 hours of life, whereas of the 61 who had cord nRBC <3.5%, 5 (8.2%) developed hyperbilirubinemia after 72 hours of life (Table 6).

**Table 6:** Cord nucleated red blood cell counts and serum bilirubin (n=123) The p-value is 0.001*, which is statistically significant.

Nucleated red blood cells %	Serum bilirubin after 72 hours ≥12 mg/dl n (%)	Serum bilirubin after 72 hours <12 mg/dl n (%)	p-value
≥3.5 (n=62)	28 (45.2)	34 (54.8)	<0.001*
<3.5 (n=61)	05 (08.2)	56 (91.8)	
Total	33	90	

Multiple linear regression was done to predict bilirubin at 72 hours of life based on cord albumin levels, bilirubin, nRBC, and reticulocyte count. A significant regression equation was found [F(4,118)=23.341, p<0.001, with an R2 of 0.442]. Newborn’s predicted serum bilirubin at 72 hours is equal to 10.663-0.009 (cord albumin) + 0.05 (cord bilirubin) + 0.57 (cord NRBC) + 0.18 (cord retic count), with an error of ±1.973. Cord nRBC and cord reticulocyte count were significant predictors, and cord albumin and cord bilirubin were not significant (Tables 7-9).

**Table 7:** Model summary

Model	R	R^2^	Adjusted R^2^	RMSE
H_0_	0.000	0.000	0.000	2.597
H_1_	0.665	0.442	0.423	1.973

**Table 8:** Analysis of variance Note: The intercept model is omitted, as no meaningful information can be shown.

Model		Sum of squares	df	Mean Square	F-value	p-value
H_1_	Regression	363.471	4	90.868	23.341	<0.001
	Residual	459.376	118	3.893		
	Total	822.847	122			

**Table 9:** Multiple regression analysis

Model		Unstandardized	Standard error	Standardized	t-value	p-value	95% CI	
							Upper	Lower
H_0_	(Intercept)	10.199	0.234		43.211	<0.001	9.655	10.582
H_1_	(Intercept)	10.663	3.130		3.407	<0.001	4.464	16.862
	Cord albumin	−0.947	0.756	−0.092	−1.253	0.213	−2.444	0.550
	Cord bilirubin	0.299	0.433	0.050	0.690	0.491	0.558	1.156
	Cord nRBC	0.374	0.048	0.570	7.789	<0.001	0.279	0.469
	Cord Retic Count	0.214	0.086	0.187	2.486	0.014	0.043	0.384

**Figure 1:**
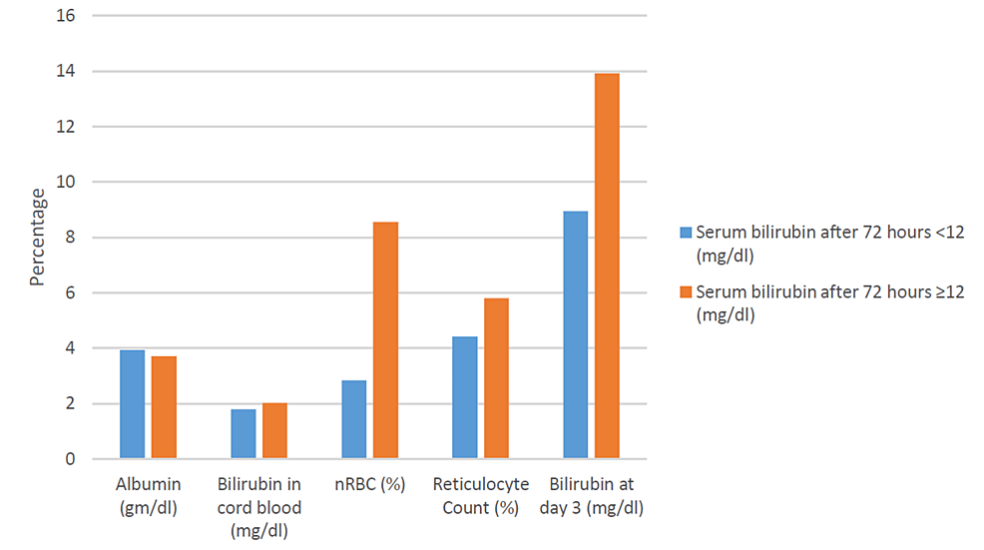
Shows the comparison of mean variables according to hyperbilirubinemia

A comparison of various variables with hyperbilirubinemia after 72 hours of life is shown in Figure 1.

## Discussion

During the first week of life, jaundice occurs in approximately 50% of term neonates and 80% of preterm neonates [1] due to the activation of mechanisms for subsequent jaundice in late fetal life. Therefore, cord albumin reticulocyte count and nRBC were examined in this study to predict hyperbilirubinemia at 72 hours of life.

In this study (Table 3), the cord bilirubin level of ≥1.75 mg/dL had a sensitivity of 69.7% and the highest negative predictive value of 82.5%, implying that neonates with low cord bilirubin levels have a lower chance of developing hyperbilirubinemia after 72 hours of life. The study also suggests that measuring cord serum bilirubin can identify neonates who are unlikely to require additional assessment or intervention.

Alaa Eldin et al. found that a total cord blood bilirubin level of ≥1.85 mg/dL in term newborns may require phototherapy, and a level of ≥2.15 mg/dL may be needed in these infants. Sun et al. suggested that cord blood bilirubin levels can predict significant hyperbilirubinemia, with a positive predictive value of 45.68% and a sensitivity of 68.27% at a level of ≥2 mg/dL [10].

A study done by Sun et al. found that cord blood bilirubin can predict the development of significant hyperbilirubinemia. In this study, cord blood bilirubin levels of ≥2 mg/dl had a positive predictive value of 45.68% and a sensitivity of 68.27 (P<0.001) [11].

Pradhan et al. observed in their study that a cord bilirubin level ≥2.5 mg/dl has a sensitivity of 84.1%, a specificity of 88.5%, a positive predictive value of 98%, and a negative predictive value of 45.1% for predicting the risk of developing hyperbilirubinemia [12].

The cord albumin level below 3.75 g/dL had a sensitivity of 54.5%, with a high negative predictive value of 50.0% and a low positive predictive value of 19.4% (Table 4). In contrast, 80.6% of newborns with cord albumin levels of ≥3.75 mg/dL did not experience hyperbilirubinemia (>12 mg/dl) after 72 hours of life, indicating that high cord albumin levels may be associated with low bilirubin levels. The current study suggests that measuring cord serum albumin can identify neonates who are unlikely to require additional testing or treatment.

Aiyappa et al. showed that a cord albumin level of 2.8 gm/dL had a sensitivity of 71.8%, a positive predictive value of 38.9%, and a negative predictive value of 88.2% [13]. In another study by Khairy et al., 67.9% of newborns who developed significant hyperbilirubinemia had a low cord serum albumin level of <2.8 mg/dl, while only 7% had levels over 3.3 mg/dL [14].

In the term group, newborns with cord albumin 2.8 g/dL developed neonatal hyperbilirubinemia in 61.2% of cases, according to Reshad et al. 6.5% of infants with cord serum albumin levels greater than 3.4 g/dL experienced substantial neonatal hyperbilirubinemia. In contrast, 32.3% of neonates had levels between 2.8 and 3.4 g/dL [15].

In this study, the cord reticulocyte level of 4.95% had a sensitivity of 60.6%, with the highest negative predictive value of 81.2% and the lowest positive predictive value of 37.0% (Table 5). However, 18.8% of newborns with cord reticulocyte counts less than 4.95% developed jaundice, indicating that low reticulocyte counts did not entirely prevent hyperbilirubinemia. The study suggests that measuring cord reticulocytes can identify newborns who are unlikely to require further assessment or intervention.

In our study (Table 6), the cord nRBC of ≥3.5% had a high sensitivity (84.8%), and this essential nRBC level had a high (91.8%) negative predictive value and a reasonably low (45.2%) positive predictive value. 8.2% of neonates with a cord nRBC of less than 3.5% had jaundice, indicating that the nRBC of less than 3.5% did not entirely prevent the development of hyperbilirubinemia. The current study’s 91.8% negative predictive value implies that measuring cord nRBC can help identify neonates who are unlikely to need further assessment or intervention.

Increased levels of nRBC and reticulocyte have been linked to conditions like stress, the use of Pitocin during labor, acute and chronic asphyxia, preeclampsia and diabetes mellitus, maternal smoking, different types of anemia, and embryonic hemolysis, according to the study by Hermanson et al. of nRBC in fetuses and infants in 2001 [16].

Very few studies show the relationship between cord nRBC and hyperbilirubinemia in neonates. The study conducted by Orhon et al. found that the cord nRBC ≥5.5 had high sensitivity (86%) and high negative predictive value (88%) [17].

Overall, our study suggests that measuring cord serum bilirubin, albumin, reticulocyte, and nucleated red blood cell levels can help predict the risk of hyperbilirubinemia in neonates. The findings are consistent with previous studies investigating the role of these biomarkers in predicting neonatal hyperbilirubinemia.

The results indicate that a cord bilirubin level ≥1.75 mg/dL, a cord albumin level below 3.75 g/dL, a cord reticulocyte level of 4.95%, and a cord nRBC level of ≥3.5% can serve as useful predictive tools for identifying neonates at risk of developing hyperbilirubinemia after 72 hours of life.

It is important to note that although these biomarkers can be useful in predicting hyperbilirubinemia, they should not be used as the sole diagnostic tool. Other clinical and laboratory factors should also be considered when making treatment decisions.

Further studies are needed to confirm our findings and explore each biomarker’s optimal cutoff values. In addition, larger studies involving a more diverse population of neonates are needed to evaluate the generalizability of our findings.

## Conclusions

In conclusion, our study adds to the growing body of evidence supporting the use of cord serum biomarkers as a valuable tool in predicting neonatal hyperbilirubinemia. Clinicians should consider measuring cord serum bilirubin, albumin, reticulocyte, and nRBC levels in neonates at risk of developing hyperbilirubinemia to identify those unlikely to need further assessment or intervention.
